# Daratumumab-based salvage therapy enables umbilical cord blood transplantation in multiline refractory, elderly T-lymphoblastic lymphoma: a case report

**DOI:** 10.3389/fimmu.2026.1743398

**Published:** 2026-02-13

**Authors:** Qian Yang, Lei Yang, Ping Cai, Yong-hui Ji, Jing-dong Zhou, Jun Qian

**Affiliations:** 1 Department of Hematology, The Affiliated People’s Hospital of Jiangsu University, Zhenjiang, Jiangsu, China; 2 Institute of Hematology, Jiangsu University, Zhenjiang, Jiangsu, China; 3 Zhenjiang Clinical Research Center of Hematology, Zhenjiang, Jiangsu, China; 4 The Key Lab of Precision Diagnosis and Treatment of Zhenjiang City, Zhenjiang, Jiangsu, China

**Keywords:** case report, daratumumab, elderly, refractory, T-lymphoblastic lymphoma, umbilical cord blood transplantation

## Abstract

While patients with T-lymphoblastic lymphoma (T-LBL) now generally have a favorable prognosis, with 3-year event-free survival rate approaching 69.2%, refractory T-LBL in older adults is almost invariably fatal, exhibiting a dismal 5-year overall survival rate of only 4%. This poor prognosis is exacerbated by frequent exclusion from cellular therapies like CD7 CAR T-cell trials. We report a case of a 60-year-old man with multi-refractory T-LBL exhibiting a partial response to hyper-CVAD followed by progression on venetoclax plus azacitidine. This patient achieved complete remission after a single cycle of DMPD salvage therapy comprising daratumumab, liposomal mitoxantrone, pegaspargase and dexamethasone. This readily accessible regimen circumvented the manufacturing delays and prohibitive costs associated with CAR T-cell platforms. It successfully bridged the patient to double umbilical cord blood transplantation, resulting in full donor chimerism by day +21 and sustained remission despite post-transplant complications. The remarkable efficacy observed in this refractory T-LBL case, contrasting sharply with historical treatment outcomes, suggests that the DMPD regimen may serve as both an immediately actionable and potentially definitive therapeutic approach for elderly patients who are ineligible for hematopoietic stem cell transplantation.

## Introduction

T-cell lymphoblastic lymphoma (T-LBL) is a rare but highly aggressive neoplasm, characterized by diffuse infiltration of T-lymphoblasts into the mediastinum, bone marrow (BM), and central nerve system (CNS) (1). It is similar with T-cell acute lymphoblastic leukemia (T-ALL) except for the latter has > 25% bone marrow blasts (2). Although the prognosis of adult T-LBL has been improved during the past 10 years due to the introduction of pediatric-Like ALL therapy — achieving CR/CRu in 90.8% of patients and 3-year rates of 69.2% overall survival (OS) — those patients over 50 years of age remain poorer in response and outcome with 5-year OS rate as low as 26% (3–5). The outcome remains dismal once the disease is relapsed, with median survival of 7.1 to 8 months and 2-year OS rate is merely 23% (6, 7). There are few therapeutic salvage options for refractory or relapsed (R/R) patients. New hope has been brought for R/R T-LBL patients by novel agents such as nelarabine, venetoclax and daratumumab (8, 9). However, nelarabine is not yet available in China, while venetoclax combinations demonstrate inconsistent efficacy due to dynamic shifts in apoptotic dependencies (10).

Herein, we report a case of a 60-year-old man with refractory T-LBL who achieved CR after one cycle of daratumumab combined with liposomal mitoxantrone, pegaspargase and dexamethasone, and then was successfully bridged to umbilical cord blood transplantation (UCBT).

## Case presentation

**Figure 1:**
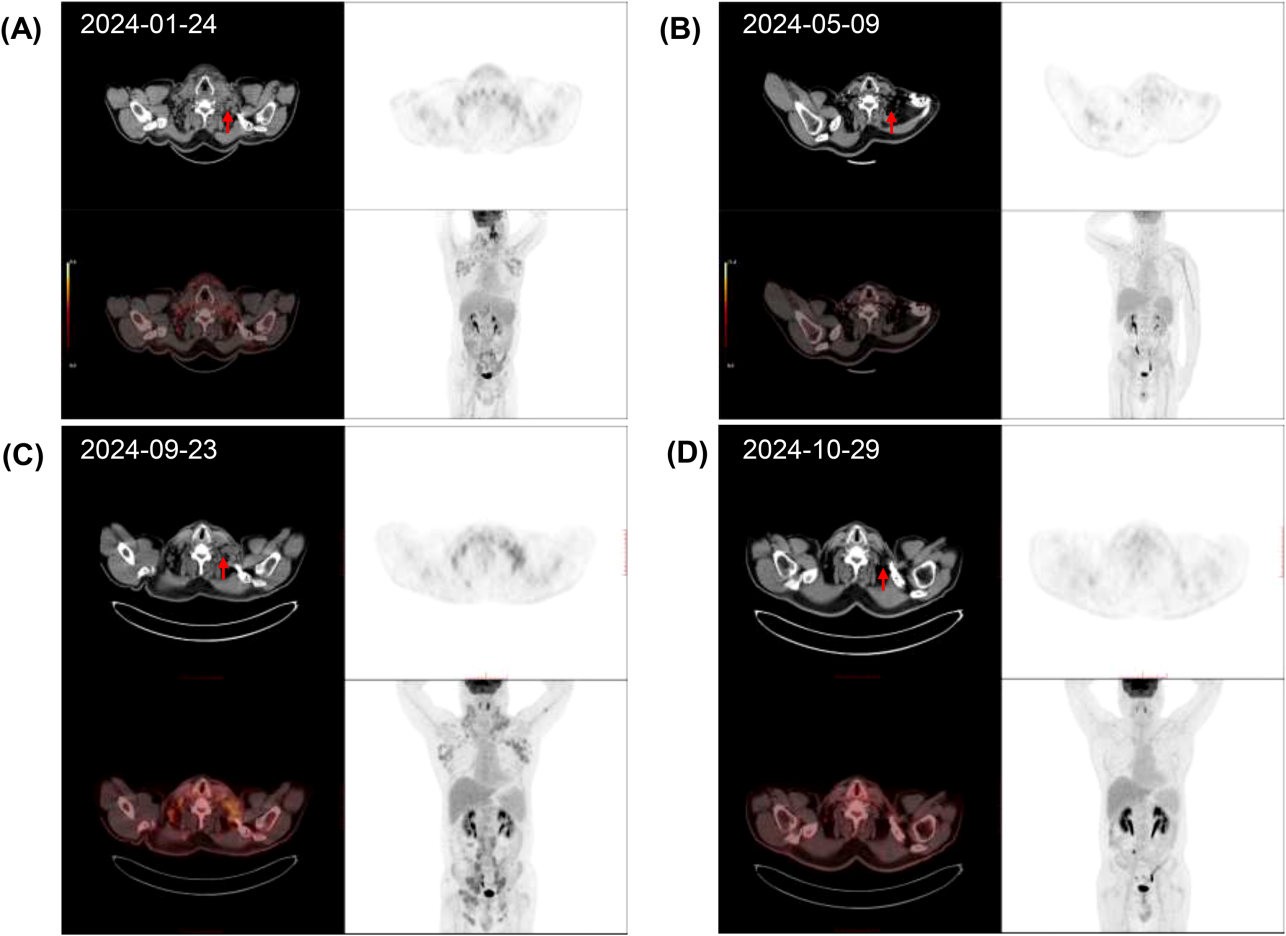
Dynamic evaluation of enlarged lymph nodes using PET-CT and CT. Enlargement and reduction of lymph nodes at initial diagnosis **(A)**, PR **(B)**, PD **(C)**, and CR **(D)** by PET-CT evaluation. The red arrows indicate the lymph nodes.

A 59-year-old male patient was admitted to other hospital on 26 December 2023, due to multiple enlarged lymph nodes and night sweats for several days. Physical examination showed enlarged lymph nodes in the neck, bilateral clavicle areas, and axilla. Positron emission tomography-computed tomography (PET-CT) revealed multiple enlarged lymph nodes in the neck, bilateral clavicle areas, axilla, mediastinum, abdominal cavity, and retroperitoneum with increased 18F-Fluorodeoxyglucose (18F-FDG) uptake (**Figure 1A**). Laboratory examination revealed pancytopenia: hemoglobin 8.9 g/dL, white blood cell count (WBC) 1.56×10^9^/L, and platelet count 68×10^9^/L. Peripheral blood smear showed 6% blasts and 2% atypical lymphocytes. Blood *Epstein-Barr* virus DNA was negative, and serum lactate dehydrogenase was normal. Bone marrow (BM) aspirate showed 5.2% lymphoblasts. Flow cytometric immunophenotyping of BM for leukemia-associated immunophenotypes (LAIP) showed positive for membrane CD3, cytoplasmic CD3, CD7, CD34, and CD2, with negative CD5, CD48, and CD99. BM biopsy demonstrated normal cellular with infiltration of immature cells with positive CD34 and negative CD3. Left neck lymph node biopsy and immunohistochemistry demonstrated T-LBL with positive stains of terminal deoxynucleotidyl transferase (TdT), CD4, CD7, CD43, and LMO2, and with Ki-67 positivity in approximately 70% of cells. Weak positivity was observed for CD3, CD5, and PAX-5. CD2, CD8, CD19, CD21, and AE1/AE3 were negative. Chromosomal analysis showed normal karyotype. Based on the comprehensive findings, the patient was diagnosed with T-LBL (IV, B, IPI 2) in the hospital outside. Four cycles of modified hyper-CVAD scheme were administered from 30 January 2024. Intrathecal injection of methotrexate, cytarabine, and dexamethasone was performed, without sign of central nervous system (CNS) disease infiltration. However, only partial remission (PR) was obtained (**Figure 1B**). The regimen of venetoclax combined with azacitidine (VA) were further given. Measurable residual disease (MRD) of BM before the second cycle of VA regimen identified 0.63% of T lymphoblasts with positive CD34, CD38, CyCD3, CD7, CD5, CD117 and CD13 (**Supplementary Figure 1**). Unfortunately, BM aspiration still showed 8.5% blasts with obvious pancytopenia after three more cycles. Furthermore, PET-CT scan revealed that the number and size of infiltrated lymph nodes significantly increased (**Figure 1C**), indicating the disease progressed. The patient was referred to our hospital for further treatment. Routine blood test showed WBC 0.22 × 10^9^/L, ANC 0.16 × 10^9^/L, Hb 5.5 g/dL, and platelet count 37 × 10^9^/L.

**Figure 2:**
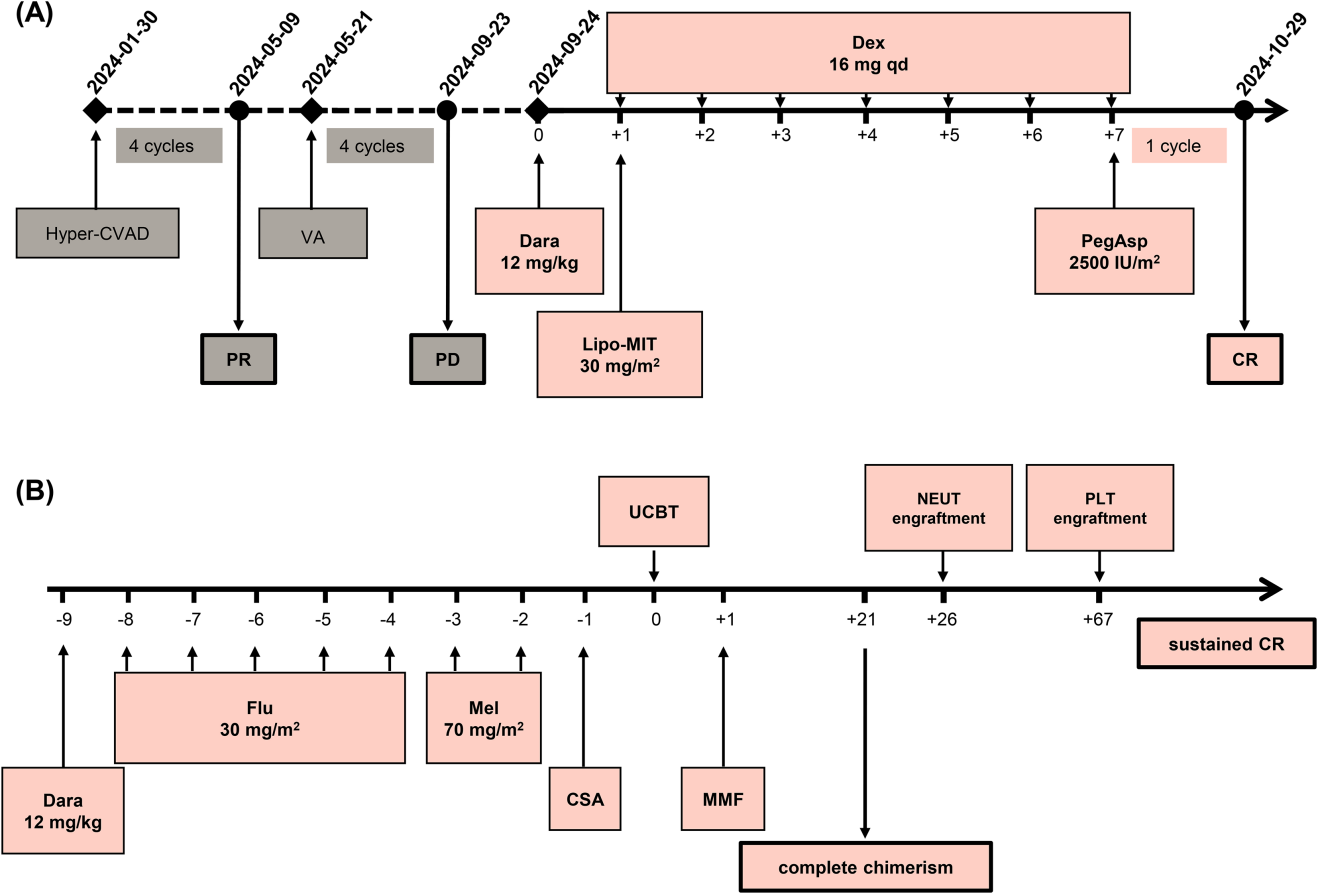
The timeline of DMPD salvage regimen treatment and UCBT process and in this case. **(A)** Hyper-CVAD: hyper-fractionated cyclophosphamide, vincristine, doxorubicin, and dexamethasone; VA, venetoclax and azacitidine; Dara, daratumumab; Lipo-MIT, mitoxantrone liposomal; Dex, dexamethasone; PegAsp, pegaspargase; PR, partial response; PD, progressive disease; CR, complete remission. **(B)** Dara, daratumumab; Flu, fludarabine; Mel, melphalan; CSA, cyclosporin; MMF, mycophenolate mofetil; UCBT, umbilical cord blood transplantation; NEUT, engraftment: neutrophil engraftment; PLT, engraftment: platelet engraftment.

Based on the documented ubiquitous expression of CD38 on T-lymphoblasts which was also confirmed in our case (11), a DMPD salvage regimen (**Figure 2A**), composed of daratumumab (12 mg/kg, day 0), liposomal mitoxantrone (30 mg/m^2^, day 1), pegaspargase (2500 IU/m^2^, day 7), and dexamethasone (16 mg qd, days 1 to 7), was administered from 24 September 2024. After one cycle of this treatment, complete remission (CR) was obtained, (**Figure 1D**) while MRD of BM assessed by FCM turned negative. There was no obvious treatment emergent adverse event occurred. Then, UCBT was subsequently proceeded, using the conditioning regimen of DFM (daratumumab 12mg/kg day -9, fludarabine 30 mg/m^2^ from days -8 to -4, melphalan 70 mg/m^2^ for days -3 and -2). Acute graft-vs-host disease (aGVHD) was prevented with mycophenolate mofetil and cyclosporin. Two units of mismatched unrelated UCBs (8/10 and 7/10 matched, respectively) were transfused on day 0. Short tandem repeat (STR) analysis of peripheral blood on day +21 demonstrated complete chimerism (99.63% donor-derived cells). Neutrophil and platelet engraftments were obtained on days +26 and + 67, respectively. The patient maintained sustained CR to date, although several complications occurred successively, including hemorrhagic cystitis with dysuria, skin chronic GVHD of grade 1, HHV-6 encephalitis, and herpes zoster virus reactivation. The UCBT treatment process is shown on **Figure 2B**.

## Discussion and conclusions

High-intensity chemotherapy remains the frontline treatment for children, adolescents and young T-ALL/LBL adults. For the elderly patients, the optimal treatment plan has not yet been determined. Targeted therapy is still lacking due to few available agents for both newly diagnosed and R/R patients. CD38 represents a compelling therapeutic target due to its homogeneous, stable expression on malignant T-lymphoblasts across all disease phases—newly diagnosed, minimal residual disease positive, refractory, and relapsed, preventing loss of patient response to daratumumab (11). Preclinically, daratumumab eliminates T-lymphoblasts via direct-on tumor, and apoptosis induction, mechanisms independent of BCL-2 inhibition pathways (12, 13). Clinically, the DELPHINUS trial demonstrated daratumumab’s activity in pediatric R/R T-ALL/LBL, with a 30% CR rate after one cycle and successful HSCT bridging in 30% of T-LBL patients (14). However, evidence remained absent for patients over 60 years with sequential multi-agent chemotherapy failure (15), underscoring the novelty of this case. Although nelarabine is used as salvage therapy for R/R T-LBL, its efficacy remains suboptimal, with reported 1-year OS rates ranging from only 37% to 52.4% (9, 16). Furthermore, the accessibility of nelarabine in China has not been established. On the other hand, venetoclax-based regimen is an effective approach to R/R T-LBL (17), Yahia et al. reported a relapsed 65-year T-LBL case that achieved CR with one cycle of venetoclax combined with the CHG regimen (18). However, BH3 profiling in ALL demonstrates that during venetoclax treatment, BCL-2 dependence shifts to BCL-xL or BCL-2/BCL-xL dependence, thereby driving venetoclax resistance (10). This may explain why our patient progressed after underwent four courses of the VA regimen. While CD7-directed CAR T-cell therapy achieves high remission rates, pivotal trials excluded patients >47 years (19). Crucially, daratumumab offers immediate accessibility as an approved off-the-shelf agent, bypassing CAR T-cell manufacturing delays (typically 3–6 weeks), logistical complexity, and high costs. The salvage therapies are listed in **Table 1**.

**Table 1:** Salvage regimens and outcomes in R/R T-LBL.

References and year	Classification	Regimen	Outcomes
Shi H et al (2024) (8)	A single-arm, open-label, and phase I study	Dara and Ven combined with CAGE regimen: Up to 2 cycles (28-day: Day 0, Dara 12 mg/kg; Days 0-7, Ven 100 mg+ G-CSF 150 μg daily; Days 1-7, cytarabine 25 mg/m² + aclarubicin 7.5 mg/m² + etoposide 25 mg/m² daily.	57.1% (ORR), 47.6% (CR)
Candoni A et al (2019) (9)	A multicenter retrospective study	Nelarabine based regimens: At least one cycle (21-day) of nelarabine at standard dose (1500 mg/m²/day, days 1, 3, 5), as monotherapy or in combination.	37% (1-year OS), 18% (5-year OS)
Shimony S et al (2023) (16)	A multicenter retrospective study	Nelarabine based regimen: At least one cycle (21-day), pediatric 650 mg/m²/day for 5 days; adult 1500 mg/m²/day, days 1, 3, and 5), as monotherapy or in combination.	52.4% (1-year OS), 37.6% (2-year OS)
Pullarkat VA et al (2021) (10)	A phase I study	Ven based regimen: venetoclax 400 mg + navitoclax at three dose levels:25 mg (≥45 kg), 50 mg (≥45 kg); 25 mg (20 to <45 kg), 100 mg (≥45 kg); 50 mg (20 to<45 kg)	66.7% (1-year OS)
Bhatla T et al (2024) (14)	A phase II study	Dara based regimen: Dara (16mg/kg) combination	20% (2-year OS), 50% (2-year RFS)
Cerrano M et al (2022) (15)	A multicenter retrospective study	Dara based regimens: Dara 16 mg/kg weekly ×8, then Q2W ×8, then monthly until PD (monotherapy or combination)	20% (ORR)
Lu P et al. (2022) (19)	A phase I trial	CD7 CAR-T therapy: NS7CAR T cells: 0.5, 1–1.5, or 2 × 10^6^ cells/kg (single infusion)	Day 28: 95% CR/CRi (19/20; all are BM MRD negative.
Guan W et al. (2020) (26)	A phase I/II trial	Chi based regimen: Chi combination for 2 cycles	54.2% ± 16.2% (2-year PFS)

Ven, venetoclax; Dara, daratumumab; Q2W, every 2 weeks; ORR, objective remission rate; CR, complete remission; OS, overall survival; RFS, relapse-free survival; CAR T therapy, chimeric antigen receptor T-cell therapy; NS7CAR T, naturally selected 7CAR; CRi, complete remission with incomplete hematologic recovery; BM, bone marrow; MRD, minimal residual disease; Chi, chidamide; PFS, progression-free survival.

The DMPD salvage regimen leveraged critical synergistic properties: Liposomal mitoxantrone enhanced lymphoid malignancy penetration while potentially mitigating cardiotoxicity (20), pegaspargase exploited metabolic vulnerabilities through asparagine depletion, disrupting protein synthesis in lymphoblasts (21), daratumumab provided immunomodulation and direct tumor targeting, independent of apoptotic dependencies compromised in venetoclax-resistant disease (22). Dexamethasone, a cornerstone of most therapeutic regimens for lymphoid malignancies in adults, exerts its effects through growth arrest, induction of programmed cell death, and mitigation of chemotherapy-related side effects (23). This combination approach achieved rapid tumor debulking within a single cycle, indicating a faster response than the daratumumab plus bortezomib and dexamethasone regimen in another R/R T-LBL case (24), and greater efficacy than daratumumab plus nelarabine salvage therapy in another case (25).

The DMPD regimen demonstrated manageable toxicity in this elderly patient. No treatment-related mortality (TRM) occurred, contrasting with the 11.8% TRM observed in some salvage regimens (26). This study has several important limitations inherent to its design as a single-center case report. First and foremost, the experience of a single patient cannot be generalized to establish the safety, efficacy, or optimal dosing of the DMPD regimen for the broader population with R/R T-LBL. The observed favorable outcome may be influenced by unique patient characteristics, including disease biology, prior treatment history, and overall fitness. Furthermore, the retrospective nature of the analysis means that data collection was not prospective or protocol-defined, which may introduce reporting bias. Despite these limitations, our findings warrant further investigation. Future prospective studies should systematically validate CD38 and other biomarkers, optimize chemotherapy backbones to reduce toxicity, and explore rational combinations, such as with BCL-xL inhibitors for venetoclax-resistant disease. Crucially, clinical trials should prioritize the inclusion of elderly patients, who constitute one-third of T-LBL cases yet remain underrepresented in current studies (4, 27).

Hematopoietic stem cell transplantation (HSCT) is an essential intervention for R/R T-LBL, significantly improving prognosis compared to patients not receiving this treatment (7). Moreover, in a T-LBL cohort study, the allo-PBSCT group demonstrated significantly higher 2-year OS and PFS compared to the non-SCT group (27). Rapid availability with no risk to the donor, low immunogenicity and less HLA-match stringency of umbilical cord blood expand donor options for elderly patients (28), thus serving as a viable alternative for critically ill seniors requiring emergency transplantation, evidenced by 100% donor chimerism by day 21 post-transplant. Prior daratumumab may interfere with the indirect antiglobulin test (IAT) of blood typing. To minimize the requirement for red blood cell transfusions during transplant, several considerations must be taken into account. Firstly, it is advisable to choose donor stem cells of the same blood type as much as possible. Secondly, hemoglobin levels can be increased before transplantation if high blood transfusion demand is expected. Moreover, corresponding methods should be used to block the binding of anti-CD38 antibody with CD38 molecule and eliminate this interference, such as treating reagent RBCs with dithiothreitol (DTT), if a patient requiring blood transfusion shows signs of hemolysis (29). Lastly, erythropoiesis-stimulating agents may be used off-label in the early stages of hematopoietic reconstitution. Meanwhile, the two major risks (transplant failure and increased TRM) of UCBT appear to have been avoided in this case (28), potentially attributable to daratumumab’s immunomodulatory properties facilitating prompt engraftment. Despite the occurrence of transplant-associated complications, the patient attained sustained remission, defying the documented 5-year OS rate of only 4% in historical cohorts with comparable disease characteristics (6).

We report the first published successful use of daratumumab-based chemoimmunotherapy as salvage therapy enabling UCBT in a 60-year-old with refractory T-LBL, this strategy overcame age-related therapeutic exclusion and achieved rapid, deep remission where conventional hyper-CVAD and novel agents (VA) failed. Additionally, it bypassed the clinical obstacle of nelarabine inaccessibility in China currently, providing a viable alternative. It offers a clinically accessible blueprint for bridging high-risk elderly patients to curative transplantation and underscores CD38’s therapeutic relevance in T-cell malignancies beyond multiple myeloma.

## Data Availability

The original contributions presented in the study are included in the article/**Supplementary Material**. Further inquiries can be directed to the corresponding authors.

## References

[1] Intermesoli T Weber A Leoncin M Frison L Skert C Bassan R . Lymphoblastic lymphoma: a concise review. Curr Oncol Rep. (2022) 24:1–12. doi: 10.1007/s11912-021-01168-x, PMID: 35059993

[2] Arber DA Orazi A Hasserjian R Thiele J Borowitz MJ Le Beau MM . The 2016 revision to the World Health Organization classification of myeloid neoplasms and acute leukemia. Blood. (2016) 127:2391–405. doi: 10.1182/blood-2016-03-643544, PMID: 27069254

[3] Blennerhassett R Kwan J Coyle L Wong K Greenwood M . Adult B- and T-lymphoblastic lymphoma treated with a paediatric acute lymphoblastic leukaemia regimen have excellent outcomes-a short report from two Sydney centres. Br J Haematol. (2020) 191:e58–60. doi: 10.1111/bjh.16998, PMID: 32720705

[4] El-Fattah MA . Prognostic factors and outcomes of adult lymphoblastic lymphoma in the United States. Clin Lymphoma Myeloma Leuk. (2017) 17:498–505.e6. doi: 10.1016/j.clml.2017.05.016, PMID: 28647403

[5] Lepretre S Touzart A Vermeulin T Picquenot JM Tanguy-Schmidt A Salles G . Pediatric-like acute lymphoblastic leukemia therapy in adults with lymphoblastic lymphoma: the GRAALL-LYSA LL03 study. J Clin Oncol. (2016) 34:572–80. doi: 10.1200/JCO.2015.61.5385, PMID: 26644537

[6] Chen H Qin Y Yang J Liu P He X Zhou S . Dismal outcome of relapsed or primary refractory adult T-cell lymphoblastic lymphoma: A retrospective study from China. Asia Pac J Clin Oncol. (2022) 18:e87–95. doi: 10.1111/ajco.13562, PMID: 34161657

[7] Samra B Alotaibi AS Short NJ Khoury JD Ravandi F Garris R . Outcome of adults with relapsed/refractory T-cell acute lymphoblastic leukemia or lymphoblastic lymphoma. Am J Hematol. (2020) 95:E245–e7. doi: 10.1002/ajh.25896, PMID: 32501545

[8] Shi H Yang F Cao M Xu T Zheng P Guo Y . Daratumumab and venetoclax combined with CAGE for late R/R T-ALL/LBL patients: Single-arm, open-label, phase I study. Ann Hematol. (2024) 103:2993–3004. doi: 10.1007/s00277-024-05775-z, PMID: 38662205

[9] Candoni A Lazzarotto D Ferrara F Curti A Lussana F Papayannidis C . Nelarabine as salvage therapy and bridge to allogeneic stem cell transplant in 118 adult patients with relapsed/refractory T-cell acute lymphoblastic leukemia/lymphoma. A CAMPUS ALL study. Am J Hematol. (2020) 95:1466–72. doi: 10.1002/ajh.25957, PMID: 32777149

[10] Pullarkat VA Lacayo NJ Jabbour E Rubnitz JE Bajel A Laetsch TW . Venetoclax and navitoclax in combination with chemotherapy in patients with relapsed or refractory acute lymphoblastic leukemia and lymphoblastic lymphoma. Cancer Discov. (2021) 11:1440–53. doi: 10.1158/2159-8290.CD-20-1465, PMID: 33593877 PMC9533326

[11] Tembhare PR Sriram H Khanka T Chatterjee G Panda D Ghogale S . Flow cytometric evaluation of CD38 expression levels in the newly diagnosed T-cell acute lymphoblastic leukemia and the effect of chemotherapy on its expression in measurable residual disease, refractory disease and relapsed disease: an implication for anti-CD38 immunotherapy. J Immunother Cancer. (2020) 8:e000630. doi: 10.1136/jitc-2020-000630, PMID: 32439800 PMC7247386

[12] Bride KL Vincent TL Im SY Aplenc R Barrett DM Carroll WL . Preclinical efficacy of daratumumab in T-cell acute lymphoblastic leukemia. Blood. (2018) 131:995–9. doi: 10.1182/blood-2017-07-794214, PMID: 29305553 PMC5833263

[13] McKeage K . Daratumumab: first global approval. Drugs. (2016) 76:275–81. doi: 10.1007/s40265-015-0536-1, PMID: 26729183

[14] Bhatla T Hogan LE Teachey DT Bautista F Moppett J Velasco Puyó P . Daratumumab in pediatric relapsed/refractory acute lymphoblastic leukemia or lymphoblastic lymphoma: the DELPHINUS study. Blood. (2024) 144:2237–47. doi: 10.1182/blood.2024024493, PMID: 39158071

[15] Cerrano M Bonifacio M Olivi M Curti A Malagola M Dargenio M . Daratumumab with or without chemotherapy in relapsed and refractory acute lymphoblastic leukemia. A retrospective observational Campus ALL study. Haematologica. (2022) 107:996–9. doi: 10.3324/haematol.2021.279851, PMID: 35021604 PMC8968887

[16] Shimony S Liu Y Valtis YK Paolino JD Place AE Brunner AM . Nelarabine combination therapy for relapsed or refractory T-cell acute lymphoblastic lymphoma/leukemia. Blood Adv. (2023) 7:1092–102. doi: 10.1182/bloodadvances.2022008280, PMID: 36508268 PMC10111357

[17] Cao H-Y Chen L-L Wan C-L Hu X-H Wu B Yang L . Venetoclax combined with azacitidine was effective and safe for relapsed/refractory T-cell acute lymphoblastic leukemia/lymphoblastic lymphoma: preliminary results of a phase 2, multicenter trial. Blood. (2023) 142:1501. doi: 10.1182/blood-2023-179733 37917081

[18] Zhou M Yang Y Zhang X Jiao Y Guo Z . Combination of venetoclax with CHG regimen in refractory/relapsed T-lymphoblastic lymphoma/acute lymphoblastic leukemia: a case series and literature review. Discov Oncol. (2025) 16:1202. doi: 10.1007/s12672-025-03055-4, PMID: 40591114 PMC12214171

[19] Lu P Liu Y Yang J Zhang X Yang X Wang H . Naturally selected CD7 CAR-T therapy without genetic manipulations for T-ALL/LBL: first-in-human phase 1 clinical trial. Blood. (2022) 140:321–34. doi: 10.1182/blood.2021014498, PMID: 35500125

[20] MA J Gong T Zhao D Zhu X Yan X Ma J . Mitoxantrone hydrochloride liposome containing regimen in patients with adult acute lymphoblastic leukemia: A multicenter, retrospective, real-world study. Blood. (2023) 142:5897. doi: 10.1182/blood-2023-187864

[21] Heo YA Syed YY Keam SJ . Pegaspargase: A review in acute lymphoblastic leukaemia. Drugs. (2019) 79:767–77. doi: 10.1007/s40265-019-01120-1, PMID: 31030380 PMC6531401

[22] Saygin C Giordano G Shimamoto K Eisfelder B Thomas-Toth A Venkataraman G . Dual targeting of apoptotic and signaling pathways in T-lineage acute lymphoblastic leukemia. Clin Cancer Res. (2023) 29:3151–61. doi: 10.1158/1078-0432.CCR-23-0415, PMID: 37363966 PMC10425730

[23] Scheijen B . Molecular mechanisms contributing to glucocorticoid resistance in lymphoid Malignancies. Cancer Drug Resist. (2019) 2:647–64. doi: 10.20517/cdr.2019.29, PMID: 35582582 PMC8992511

[24] Maraglino AME Sammassimo S Lolli G Clemente A Tabanelli V Pastano R . Daratumumab plus bortezomib and dexamethasone as a bridge to allogeneic transplantation in refractory T-cell lymphoblastic lymphoma. Ann Hematol. (2025) 104:3875-3879. doi: 10.1007/s00277-025-06474-z, PMID: 40569427 PMC12334505

[25] Castellanos G Pardo L López A Cornago J López JL de Las Heras A . Daratumumab and nelarabine treatment as salvage therapy for T-lymphoblastic lymphoma: A case report. Biomedicines. (2024) 12:512. doi: 10.3390/biomedicines12030512, PMID: 38540125 PMC10968291

[26] Guan W Jing Y Dou L Wang M Xiao Y Yu L . Chidamide in combination with chemotherapy in refractory and relapsed T lymphoblastic lymphoma/leukemia. Leuk Lymphoma. (2020) 61:855–61. doi: 10.1080/10428194.2019.1691195, PMID: 31755348

[27] Yu F Niu J Yang J Hou J Hao S Liang A . Optimal timing and impact of allogeneic peripheral blood stem cell transplantation in adult T-cell lymphoblastic lymphoma: insights from a large cohort multi-center real-world study in Shanghai. Bone Marrow Transplant. (2025) 60:380–8. doi: 10.1038/s41409-024-02500-2, PMID: 39706878

[28] Sanchez-Petitto G Rezvani K Daher M Rafei H Kebriaei P Shpall EJ . Umbilical cord blood transplantation: connecting its origin to its future. Stem Cells Transl Med. (2023) 12:55–71. doi: 10.1093/stcltm/szac086, PMID: 36779789 PMC9985112

[29] Lancman G Arinsburg S Jhang J Cho HJ Jagannath S Madduri D . Blood transfusion management for patients treated with anti-CD38 monoclonal antibodies. Front Immunol. (2018) 9:2616. doi: 10.3389/fimmu.2018.02616, PMID: 30498492 PMC6249335

