# Supplementary figures and images for: Daratumumab-based salvage therapy enables umbilical cord blood transplantation in multiline refractory, elderly T-lymphoblastic lymphoma: a case report
Source: Front Immunol. 2026 Feb 13;17:1743398. doi: 10.3389/fimmu.2026.1743398 (PMC12946026; doi:10.3389/fimmu.2026.1743398)

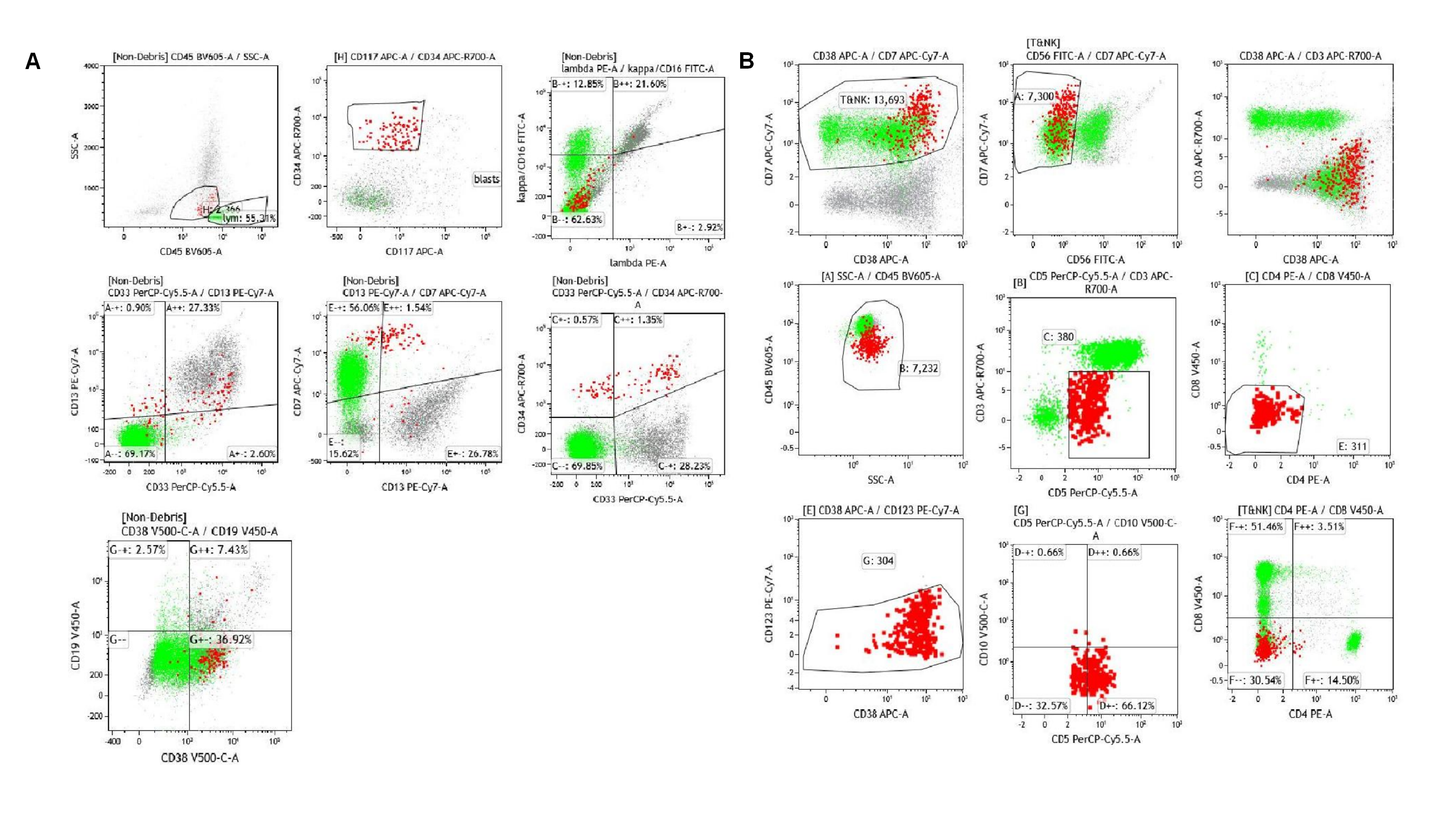

Supplement: Supplementary file 1 [file Image1.tif]
